# Evaluation of the use of video consultation in German rheumatology care before and during the COVID-19 pandemic

**DOI:** 10.3389/fmed.2022.1052055

**Published:** 2022-11-25

**Authors:** Jutta G. Richter, Gamal Chehab, Joana Reiter, Peer Aries, Felix Muehlensiepen, Martin Welcker, Hasan Acar, Anna Voormann, Matthias Schneider, Christof Specker

**Affiliations:** ^1^Policlinic for Rheumatology and Hiller Research Unit for Rheumatology, Medical Faculty, Heinrich Heine University Düsseldorf (HHUD), University Clinic, Düsseldorf, Germany; ^2^Immunologikum, Hamburg, Germany; ^3^Center for Health Services Research, Faculty of Health Sciences, Brandenburg Medical School Theodor Fontane, Rüdersdorf, Germany; ^4^MVZ für Rheumatologie Dr. Martin Welcker GmbH and RheumaDatenRhePort (rhadar), Planegg, Germany; ^5^German Society for Rheumatology, Berlin, Germany; ^6^Department of Rheumatology and Clinical Immunology, KEM Kliniken Essen-Mitte, Essen, Germany

**Keywords:** video consultancy, COVID-19, telemedicine, digital health, rheumatology

## Abstract

**Background:**

The COVID-19 pandemic led to transformations in healthcare infrastructures and increased use of (innovative) telemedicine (TM) tools. Comparison of the use of video consultation (VC) in rheumatology in the pre-pandemic period and during the pandemic might allow for evaluating this new form of consultancy in healthcare due to changing conditions and possibilities.

**Materials and methods:**

Cross-sectional nationwide online survey among German rheumatologists and rheumatologists in training between March and May 2021 promoted by newsletters and Twitter posts.

**Results:**

Results refer to 205 participants. The majority was male (59%), older than 40 years (90%). Thirty-eight percent stated to have employed TM before (“digital users”), 27% were using VC as part of their TM expertise (“VC-users”), 10% stated to have experience with TM but not VC (“TM-users”). Those negating the use of any TM (62%) were designated as “digital non-users.” TM-Knowledge was self-rated as 4 [median on a Likert Scale 1 (very high) to 6 (very low)] with a significant difference between digital users (VC-user 2.7 ± 1.2, TM-user 3.2 ± 1.1) and digital non-users (4.4 ± 1.3). The reported significant increase of VC use during the lockdown periods and between the lockdowns compared to the pre-pandemic phase was regarded as a proxy for VC acceptance in the pandemic. Reasons for VC non-use were administrative/technical efforts (21%), lack of technical equipment (15%), time constraints (12%), time required for individual VC sessions (12%), inadequate reimbursement (11%), lack of demand from patients (11%), data security concerns (9%), poor internet connection (8%), and lack of scientific evaluation/evidence (5%). Physicians considered the following clinical situations to be particularly suitable for VC: follow-up visits (VC-user 79%, TM-user 62%, digital non-user 47%), emergency consultations (VC-user 20%, TM-user 33%, digital non-user 20%), and patients presenting for the first time (VC-user 11%, TM-user 19%, digital non-user 8%).

**Conclusion:**

Even though the pandemic situation, with social distancing and several lockdowns, provides an ideal environment for the implementation of new remote care forms as VC, its use and acceptance remained comparatively low due to multiple reasons. This analysis may help identify hurdles in employing innovative digital care models for rheumatologic healthcare.

## Introduction

The use of telemedicine (TM) gained importance in recent years due to the improvement in information and communication technology (ICT). Available approaches offer synchronous and asynchronous means for communication in healthcare (1). In rheumatology novel care concepts encompass real-time, direct exchanges of information [e.g., *via* telephone and/or video consultations (VC)] and asynchronous exchanges of information including remote-patient monitoring (e.g., *via* email, ICT platforms, Apps, and/or wearables) (1–3). However, predominantly telephone consultancy has been integrated into healthcare so far (1, 4). Studies on “telerheumatology” focused mainly on depicting feasibility and patient satisfaction with various interventions e.g., tight control concepts (5, 6).

In Germany, the implementation of VC into healthcare was legally approved in 2017 (7–10). Its implementation and usage remained limited to certain situations until 2020 (11). Similar to other countries worldwide, the coronavirus (COVID-19) pandemic led to a rapid and widespread disruption of personal healthcare in Germany as attendance to clinics was significantly limited due to regulatory constraints (12), local institutional restrictions, and patients’ reluctance (13–15). TM tools rapidly and widely gained international acceptance as feasible management tools allowing for offering still some form of care for rheumatic diseases and have been adopted in position papers and guidelines for the management of rheumatic diseases in adult patients during the COVID-19 pandemic (11, 16, 17). Evaluation of the specificity and sensitivity of follow-up virtual VCs for treatment decisions has been published recently (18). Reports on physicians’ and patients’ satisfaction with VC in the pandemic are available (19, 20).

Data on the use of VCs from the physician’s perspective and in German rheumatology healthcare and its adoption in a pandemic situation are scarce (21). This study aimed to evaluate the use of VC before and during the COVID-19 pandemic including the waves of lockdowns among physicians working in rheumatological care in Germany to support identifying restrictions and obstacles in the implementation of VC in clinical practice in rheumatology.

## Materials and methods

From March 25th, 2021 until May 31st, 2021 a nationwide voluntary online survey was conducted as a cross-sectional study among German rheumatologists or physicians in training for rheumatology. The Policlinic for Rheumatology & Hiller Research Unit for Rheumatology developed the applied questionnaire in collaboration with members of the Commission “Digital Rheumatology” of the German Society for Rheumatology (DGRh), and the co-authors. Experiences of the recent study on the acceptance of telerheumatology by rheumatologists and general practitioners in Germany reported by Muehlensiepen et al. were taken into account (21). Items were assessed as single or multiple select variables from pre-given answering options or as Likert Scales from 1 to 6 according to a common German grading system.

The Checklist for Reporting Results of Internet E-Surveys (CHERRIES) was followed as much as possible (22). Thus, the online questionnaire containing 50 questions (including free-text options where necessary) was applied using adaptive questioning leading to various numbers of pages when the questionnaire was distributed. Due to content related order, items were not randomized or alternated. It was possible to leave questions unanswered; therefore, completeness checks were not included.

The survey was promoted *via* several newsletters of the DGRh and the Association of German Rheumatology Professionals (BDRh, the official association of office-based German rheumatologists). The 2021 annual meeting of the BDRh was used for advertisement. In addition, the survey was posted twice on the social media account of the Working Group “Young Rheumatologists” within the German Society for Rheumatology (#rheumadocs). The survey invitation was thus distributed to 1,650 (DGRh) and 527 (BDRh) persons, respectively, with newsletter recipients overlapping. Due to data protection issues group specific response rates cannot be estimated. Incentives were not offered to participants.

Ethical approval was obtained from the local ethic committee (local study number 2020-1207). The study was registered to the German Clinical Trials Register (Identifier DRKS00023430).

Data collection was performed anonymously utilizing the survey tool QuestionPro^1^ a “web-based software for surveys, market research and experience management” offering a direct data export as IBM SPSS files to perform statistical analyses.

According to adaptive testing, participants were grouped as follows: First, participants were divided into two groups “digital user” and “digital non-user.” Second, “digital users” who used TM (according to WHO definition as “use of ICT to improve patient outcomes by increasing access to care and medical information”) but never VC were designated as “TM user” and those who used TM and VC were grouped as “VC user” (23). TM use was self-rated by participants. The group of digital non-users denied the use of VC and other TM applications.

Statistical computations used IBM SPSS Statistics version 27. Predominantly descriptive statistics were executed. Values are expressed as valid percentages for discrete variables, or as mean ± standard deviation (SD), range, IQR or median for continuous variables. Differences in locations were tested *via* Chi Square and–where appropriate–non-parametrically (Wilcoxon signed-rank test and Kruskal–Wallis Tests). All statistical tests were performed two-tailed, *p*-values less than 0.05 were considered significant.

## Results

We registered *n* = 263 survey participants. Of these, 58 who did not answer any question were excluded. Thus, reported data refer to *n* = 205 participants, of which *n* = 128 (62%) reported themselves as digital non-users. Out of *n* = 77 (38%) digital users *n* = 56 (27% of all respondents, 73% of digital users) stated to use VC, and 21 (10%) not (TM users).

### Description of study participants

The survey obtained respondents from all over Germany and one from Austria.

Similar to distribution among DGRh members the majority of respondents was male (59%, *n* = 90/153). Most (90%, *n* = 136/151) were older than 40 years of age. Gender and age categories were not significantly different between current digital users and digital non-users. Most worked office-based (49%, *n* = 68/139), followed by clinics (41%, *n* = 57/139), and both locations (10%, *n* = 14/139). Participants usually worked in urban areas (83%, *n* = 119/144).

### Knowledge of telemedicine and perceived telemedicine scenarios

Knowledge of TM was self-rated as 4 {median on a Likert Scale [1 (very high) –6 (very low)], IQR 2.0}. Self-rated knowledge of TM was similar for current VC users (2.7 ± 1.2, median 3.0, IQR 2), and TM users 3.2 ± 1.1, median 3.0 (IQR 2.0). Both groups’ knowledge was significantly higher compared to the knowledge ratings of the digital non-users (4.4 ± 1.3, median 4.0, IQR 3.0, *p* < 0.001).

Digital users (*n* = 77) considered the use of TM applications as useful for the following scenarios: physician-physician interaction (73%, *n* = 56/77), physician-patient interaction (92%, *n* = 71/77), physician-physician assistant (31%, *n* = 24/77), physician assistant-patient (61%, *n* = 47/77). In addition to VC, users reported telephone consultation (70%, *n* = 54/77), digital health applications (14%, *n* = 11/77), and Email (4%, *n* = 3/77).

### Video consultation use pre-pandemic and throughout the pandemic

Video consultation was offered by 27% (*n* = 56/205) of all participants and 73% (*n* = 56/77) of the digital users throughout the assessed periods. Use of VC was higher in respondents working in private practices than respondents in hospitals. The initiative to offer VC was mostly taken by the physicians themselves (77%, *n* = 43/56) and rarely by employers (9%, *n* = 6/69). The initiative to conduct a VC originated from physicians (64%, *n* = 36/56) more than from patients (38%, *n* = 21/56).

**FIGURE 1:**
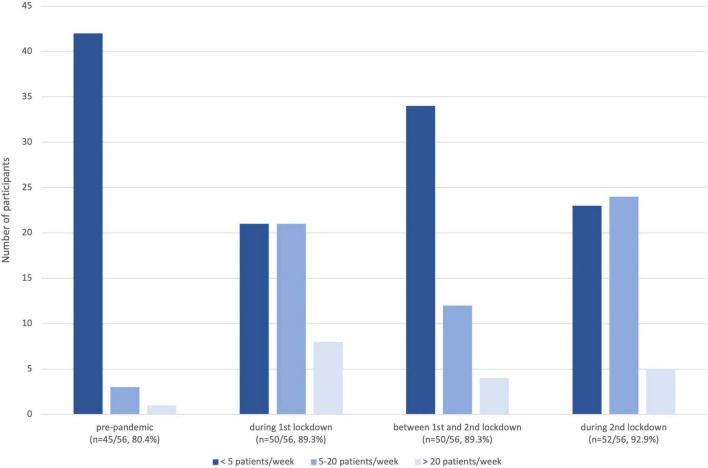
Grouped number of patients managed by participants *via* VC in the different lockdown phases.

**FIGURE 2:**
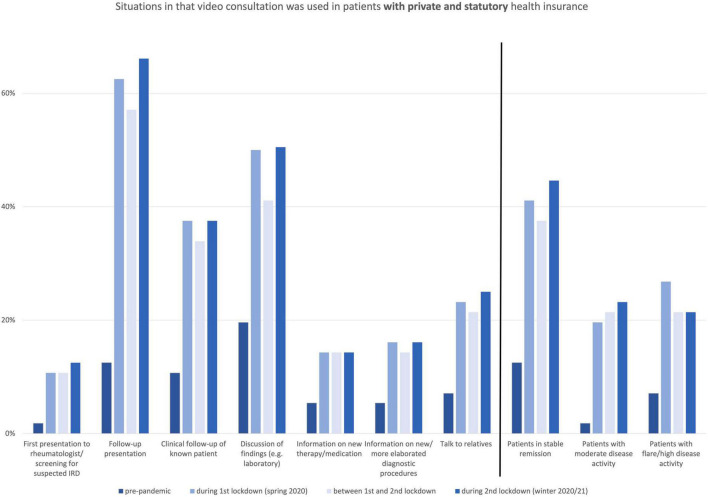
Situations in that video consultation was used in patients with private and statutory health insurance.

Figure 1 illustrates the number of participants who used the VC for how many patients in the various lockdown phases showing an increase in the use of VC during the lockdown periods. Even between the lockdown phases, VC use was higher than in the pre-pandemic phase. Figure 2 depicts for which caring situations VC was used.

### Rankings of experienced video consultations and video consultation’s rankings with respect to future use

The participants were asked whether VC could be compared to other patient communication scenarios regarding routine rheumatological care. VC was not perceived to be comparable to a face-to-face visit [mean 4.3 ± 1.3, median 4.5, *n* = 52/56, Likert Scale 1 (I agree completely)–6 (I do not agree at all)], but at least comparable to a telephone consultation [mean 3.3 ± 1.3, median 3.0, *n* = 51/56, Likert Scale 1 (I agree completely) – 6 (I do not agree at all)]. VC was regarded as an additional mean of communication with the patient [mean 2.3 ± 1.5, median 2.0, *n* = 51/56, Likert Scale 1 (I agree completely) – 6 (I do not agree at all)].

Video consultation users’ median scores of assessability of clinical changes ranged between 2 and 4, see Table 1. In the assessments for which rheumatic diseases VC could be useful, it became apparent that digital users estimated the value of VC as more than twice as high compared to digital non-users, this holds true for all diseases asked for with only slight differences between disease groups (see Table 2).

**TABLE 1:** Ratings of the assessability of clinical changes *via* VC [Likert scale 1 (I agree completely)–6 (I do not agree at all)].

	VC user (*n* = 56)
Assessment of …	Mean ± standard deviation (median)
General condition and nutritional status of the patient (*n* = 48)	2.6 ± 1.4 (2)
Hands (*n* = 48)	3.0 ± 1.4 (3)
Skin lesions (*n* = 48)	2.7 ± 1.2 (3)
Oral cavity/mouth opening (*n* = 48)	3.8 ± 1.6 (4)
Movement restrictions (e.g., dynamics when standing up, lifting arms) (*n* = 48)	3.3 ± 1.3 (3)
Eyes (*n* = 48)	3.8 ± 1.7 (4)

**TABLE 2:** Diseases and patients’ presentation scenarios for which VC was valued.

	Digital user (*n* = 77)		Digital non-user (*n* = 128)	
	First presentation % (*n*)	Follow-up presentation % (*n*)	First presentation % (*n*)	Follow-up presentation % (*n*)
Osteoarthritis	9.1 (7)	48.1 (37)	4.7 (6)	17.2 (22)
Axial spondyloarthritis	7.8 (6)	57.1 (44)	1.6 (2)	22.7 (29)
Fibromyalgia	9.1 (7)	44.2 (34)	7.0 (9)	18.8 (24)
Juvenile idiopathic arthritis	3.9 (3)	44.2 (34)	1.6 (2)	16.4 (21)
Connective tissue diseases	3.9 (3)	42.9 (33)	0.8 (1)	16.4 (21)
Crystal arthropathies	5.2 (4)	46.7 (36)	1.6 (2)	18.8 (24)
Osteoporosis	10.4 (8)	40.3 (31)	4.7 (6)	19.5 (25)
Psoriatic arthritis	5.2 (4)	57.1 (44)	1.6 (2)	21.9 (28)
Rheumatoid arthritis	6.5 (5)	59.7 (46)	2.3 (3)	21.9 (28)
Vasculitides	3.9 (3)	32.5 (25)	1.6 (2)	16.4 (21)

Additionally, future target groups, respectively, scenarios for VC were assessed to pre-given categories. Respondents valued VC more for follow-up visits (VC user 79%, *n* = 44/56, TM user 62%, *n* = 13/21, digital non-user 47% *n* = 60/128), than for emergency consultation (VC user 20%, *n* = 11/56, TM user 33%, *n* = 7/21; digital non-user 20%, *n* = 25/128), and for patients presenting for the first time (VC user 11% *n* = 6/56, TM user 19%, *n* = 4/21, digital non-user 8%, *n* = 10/128). The ratings of the value of VC in application scenarios are listed in Table 3; the users rated these worse than the digital non-users, with the exception for the rating of “clinical assessment” and “flare/high disease activity.”

**TABLE 3:** Ratings of the value of VC in pre-given application scenarios [mean ± standard deviation (*n*), Likert scale (very useful)–6 (not at all useful)].

Scenario	Digital user Mean ± standard deviation Likert scale 1 (very useful)–6 (not at all useful)	Digital non-user Mean ± standard deviation Likert scale 1 (very useful)–6 (not at all useful)
First presentation to rheumatologist/screening for suspected IRD*	4.7 ± 1.4	3.9 ± 1.7
Follow-up presentation*	2.4 ± 11.1	2.0 ± 1.2
Clinical assessment	2.7 ± 1.1	2.9 ± 1.6
Discussion of findings (e.g., laboratory)*	2.1 ± 1.3	1.6 ± 1.3
Information on new therapy/medication*	2.9 ± 1.6	1.8 ± 0.9
Information on new/more elaborated diagnostic procedures*	2.8 ± 1.6	1.9 ± 1.2
Talk to relatives	2.8 ± 1.6	2.4 ± 1.5
Stable remission*	1.9 ± 1.1	1.5 ± 1.1
Moderate disease activity	3.3 ± 1.3	3.2 ± 1.3
Flare/high disease activity	4.3 ± 1.6	4.3 ± 1.3

*Significant differences with *p*-values < 0.05 (Wilcoxon-Mann–Whitney-Test).

Out of 205 participants, 46 (34 VC users, 2 TM users and 10 digital non-users) will offer VC in the future. “Thinking about offering VC in the future” (regardless of whether VC was already performed) were *n* = 55 with the largest amount in the digital non-user group (42%, *n* = 40/96), followed by VC users (9%, *n* = 9/96), and TM users (6%, *n* = 6/96). Seventeen were undecided. Of those who offered VC already, 5 (9%) will not continue its use in the future. Of those who offer VC and those who think about it, VC will be offered during fixed time slots by 65% (*n* = 66/101), at the request of the patient by 47% (*n* = 47/101), and for emergency consultation by 14% (*n* = 14/101) of the participants (multiple response question). This distribution was not significantly different between VC, TM, and digital non-users.

### Reasons for non-use of video consultation

Participants were asked what reasons prevent them from using VC *via* a multiple selection question. Reasons that discourage TM users and digital non-users from implementing VC encompassed administrative/technical efforts (21%), lack of technical equipment (15%), time constraints (12%), time required for individual VC sessions (12%), inadequate reimbursement (11%), lack of demand from patients (11%), data security concerns (9%), poor internet connection (8%), and lack of scientific evaluation/evidence (5%).

### Technical and administrative challenges for video consultation usage

Seventy percent (*n* = 38/54) of our respondents lacked the complete necessary hardware equipment, technical challenges were “manageable” (e.g., procuring the technology). Hardware investments encompassed mostly microphones (34%, *n* = 19/56) and cameras (46%, *n* = 26/56). The resulting costs amounted to a median of 250€. The familiarization with the technology from the physician’s perspective was rated as 2.3 ± 1.2 [median 2, IQR 2, Likert scale simple (1) to difficult (6)] and for assistant personnel as 2.9 ± 1.2 [median 3, range/IQR 2, Likert scale simple (1) to difficult (6)].

## Discussion

In the present study, we report on the usage of TM with a focus on VCs by German rheumatologists during two pandemic imposed lockdown periods in 2020 and 2021. Interest in VC has been reported in 43% of rheumatologists studied by Muehlensiepen et al. (21) and the pandemic situation with social distancing and several lockdowns should theoretically have provided an ideal environment for the implementation of new remote care forms such as VC. However, in our cohort use and acceptance (27%) remained low even over time. As reported by others (24, 25) and compared to the pre-pandemic era, the number of participants using VC increased during the first lockdown phase by +9%. Use flattened between the two lockdowns but did not return to pre-pandemic values. In the second German lockdown phase, the usage of VC increased again by +4%. Our survey also revealed a certain reluctance toward the technology, (dis)comfort with technology, and inadequate reimbursements among our survey respondents representing hurdles in employing innovative digital care models for rheumatologic healthcare. VC use and increases of video-based visits were recently also conveyed by others (13, 26, 27). A study from Li et al. and the RISE registry reported a mean increase of TM visits of 12% without differentiating VC from other forms of TM (28). Although we noted a relevant increase of VC up to 20 patients per responder, the number of patients per responder served *via* VC rarely exceeded 20 throughout the period in all pandemic phases observed. This is in line with data from UK where VC use was rare as well (less than 1 in 4 consultations) (29).

From the patients’ perspective, in the first pandemic summer (June 2020) VCs were reported as an appropriate alternative in the follow-up during the COVID-19 pandemic (19). However, patients are obviously less likely to take the initiative for VCs (38% according to our survey data) and patients seem to be quite satisfied with “usual” telephone consultations that are regarded as convenient, safe, useful, and effective (30). When considering VC for remote care, it is important to consider that technological prerequisites to access VC still vary widely between younger (18–49 years) patients and those over 80 years (30).

Our respondents valued VC predominantly for follow-up visits and within fixed time slots and only rarely for emergency consultation. This is consistent with the findings from others who reported that rheumatologists were most interested in using TM applications, respectively, VC for follow-up visits (19, 21, 31). The assessability of clinical findings through VC was rated as moderate, with the best ratings for the assessability of skin lesions and hands. This likely explains the low rating of VC for emergency and other consultations, where a more comprehensive clinical evaluation is required (32). The ratings for VC’s use case scenarios (e.g. assessability of clinical findings) were significantly worse among VC users than those of non-users which may explain why some VC users are re-considering offering VC in the future.

Our reported ratings can give new VC users some guidance in their implementation processes and retain the expectations of this realistic. VC was usually not regarded as eligible for new patients. This is in line with findings from Singh et al. (13).

According to our data, VC should primarily be used as a supplement as VC is not perceived to be comparable to face-to-face visits. A review published by Sutherland et al. in palliative care—a very specific caring situation–also reported that VCs are typically no complete substitutes for face-to-face visits (33). In contrast, 57% of GPs lately considered VCs equal to or even better than face-to-face consultations (31). Recently VC has been proposed as a mean for standard tight-control strategies (18). Furthermore, physician-physician interaction for case discussion is limited in the pandemic as well. Of our respondents, 73% considered VC as a useful tool for physician-physician interaction, thus VC might be adopted also as a tool for this interaction.

Use of VC was higher in respondents working office-based than respondents working in hospitals, which is in line with other data reported from Germany (34). Lower burdens in terms of hierarchical and administrative decision-making are likely to be reasons for this although, from an economic perspective, VC and the existing remuneration arrangements provide no relevant incentive for implementation (16) even when required financial investments were at a low level. VC was performed not only during “usual business hours” which emphasizes the high level of commitment of the colleagues and might also be attributed to the fact that physicians in private practice also withdrew to home offices. The most important competitor for VC seems to be the telephone, as it is the standard of communication and has been used in teleclinic studies more often than VC (35). In-person visits are considered the “gold standard” in rheumatology care.

Knowledge of TM was self-rated low by our participants, especially among digital non-users. The data correspond to data reported (21) and depict that education on TM applications and current remote caring concepts is still warranted in rheumatologists to successfully implement these concepts in modern patient management, and to increase TM’s acceptability and safety (36). The recently published points to consider for remote care in rheumatic and musculoskeletal diseases might help in the implementation processes as well (37).

### Limitations

Although the survey was performed, advertised, and distributed online using the major and relevant online communication channels of German rheumatologists response rate ranges between 12 and 16% (depending on the amount of overlapping newsletter recipients) and was thus lower than the expected 20%. We attributed this to the fact that during the COVID-19 pandemic, at least DGRh newsletter recipients were exposed to a large number of DGRh online surveys (38). The majority of our respondents was aged 40 + and thus age-biased results can’t be excluded. The majority of respondents worked in urban areas, so our data might be location biased. However, respondents lived throughout the entire country, thus representativeness is still assumed. The decision not to use offline channels (print organs, etc.) may have led to a selection and non-response bias. Furthermore, our analysis might be influenced by respondents’ individual perceptions felt during the pandemic. We did not include patients’ experiences with VC and their preferences.

## Conclusion

Our results report low VC use in rheumatology in the COVID-19 pandemic in Germany, a country with high regulatory requirements for this form of patient management. Given the concurrent reported decline in physician face-to-face consultations (39) and the other pandemic-given restrictions, utilization as well as acceptance remained low. We revealed a wide spectrum of reasons for utilization and non-utilization of VC. The identified obstacles should be addressed by policymakers, payers legislators, medical societies, and all other stakeholders in the healthcare system to provide better foundations for future innovative care models.

## Data availability statement

The data supporting the conclusions of this article are available on reasonable request by the authors.

## Ethics statement

The studies involving human participants were reviewed and approved by the Ethics Committee of Medical Faculty at Heinrich Heine University Düsseldorf. Written informed consent for participation was not required for this study in accordance with the national legislation and the institutional requirements.

## Author contributions

JuR, GC, JoR, PA, FM, MW, AV, MS, HA, and CS designed, performed, analyzed the study, drafted the manuscript, and analyzed the data. All authors contributed to the manuscript’s revision, read, and approved the submitted version

## Footnotes

1
https://www.questionpro.de/
